# Prevalence of Themes Linked to Delayed Presentation of Breast Cancer in Africa: A Meta-Analysis of Patient-Reported Studies

**DOI:** 10.1200/JGO.19.00402

**Published:** 2020-05-21

**Authors:** Olayide S. Agodirin, Isiaka Aremu, Ganiyu A. Rahman, Samuel A. Olatoke, Halimat J. Akande, Adetunji S. Oguntola, Olalekan Olasehinde, Sheriff Ojulari, Amarachukwu Etonyeaku, Julius Olaogun, Anya Romanoff

**Affiliations:** ^1^ Department of Surgery, University of Ilorin and University of Ilorin Teaching Hospital, Ilorin, Kwara State, Nigeria; ^2^ Department of Surgery, University of Ilorin Teaching Hospital, Ilorin, Kwara State, Nigeria; ^3^ Department of Surgery, University of Cape Coast and Cape Coast Teaching Hospital, Cape Coast, Ghana; ^4^ Department of Radiology, University of Ilorin and University of Ilorin Teaching Hospital, Ilorin, Kwara State, Nigeria; ^5^ Department of Surgery, Lautech Teaching Hospital, Ogbomoso, Oyo State, Nigeria; ^6^ Department of Surgery, Obafemi Awolowo Teaching Hospital, Ile-Ife, Osun State, Nigeria; ^7^ Department of Physiology, University of Ilorin, Ilorin, Kwara State, Nigeria; ^8^ Department of Surgery, Ekiti State Teaching Hospital, Ado-Ekiti, Ekiti State, Nigeria; ^9^ Breast Surgery, Dubin Breast Center, Icahn School of Medicine, The Mount Sinai Hospital, New York, NY; ^10^ Department of Health System Design and Global Health, Icahn School of Medicine, The Mount Sinai Hospital, New York, NY

## Abstract

**PURPOSE:**

The prevalence of themes linked to delay in presentation of breast cancer (BC) and their underlying factors vary considerably throughout Africa. Regional differences and trends are largely unreported. The purpose of this research was to provide summary estimates of the prevalence and distribution of the themes and underlying factors linked to delay in the presentation of BC, regional variation, and trends in an effort to identify targets for intervention.

**DESIGN:**

We screened articles found through PubMed/Medline, African Journal OnLine, Science Direct, Google/Google Scholar, and ResearchGate. We included patient-reported surveys on the reasons linked to delayed presentation under 6 previously identified themes: symptom misinterpretation, fear, preference for alternative care, social influence, hospital-related factors, and access factors. The meta-analytical procedure in MetaXL used the quality-effect model.

**RESULTS:**

Twelve of the 236 identified articles were eligible for this review. The overall summary estimate of late presentation (> 90 days) was 54% (95% CI, 23 to 85) and was worst in the eastern and central regions. Symptom misinterpretation was the most common theme (50%; 95% CI, 21 to 56), followed by fear (17%; 95% CI, 3 to 27), hospital-related theme (11%; 95% CI, 1 to 21), preference for alternative care (10%; 95% CI, 0 to 21), social influence (7%; 95% CI, 0 to 14), and access-related theme (6%; 95% CI, 0 to 13). The most common factor underlying symptom misinterpretation was mischaracterizing the breast lesion as benign (60%; 95% CI, 4 to 100) which surpassed lack of awareness in the last decade. Misdiagnosis and failure to refer were the dominant hospital-related factors.

**CONCLUSION:**

Modifiable factors such as mischaracterizing malignant masses as benign, fear, misdiagnosis, and failure to refer were the prevalent factors contributing to delays throughout Africa. These factors are promising targets for intervention.

## INTRODUCTION

Breast cancer (BC) in Africa is most often diagnosed at an advanced stage, leading to worse outcomes. This is due, in part, to delayed presentation of symptomatic disease. Patient delay in seeking medical care is a significant barrier to effective BC management in Africa. The WHO, Breast Health Global Initiative,^1,2^ and African epidemiologists^2^ propose reduction in delay as a cost-effective strategy for improving BC outcomes in Africa. Therefore, researchers and policy makers in Africa need to understand the factors linked to delay and barriers to care to prioritize resource allocation and to plan successful interventions.

**CONTEXT**

**Key Objective**
To determine the prevalence of the themes or factors linked to delayed presentation of breast cancer (BC) in Africa and the regional variation and trends over time.
**Knowledge Generated**
Symptom misinterpretation is the most common theme linked to delayed presentation of patients with BC throughout Africa, and mischaracterizing a malignant lump as benign surpassed lack of awareness to become the most common factor underlying symptom misinterpretation in the past decade. Misdiagnosis and failure to refer were the dominant hospital-related factors.
**Relevance**
Educating the general population about the signs and symptoms of BC and encouraging them to seek medical attention if symptoms develop is a potential target for reducing late presentation of BC in Africa. Also, educating health care providers regarding triple assessment of BC symptoms is potentially a way to ensure accurate diagnosis and prompt referral for effective treatment in Africa.

The delay in presentation for health care among African patients with BC may be as long as 8 to 12 months.^3-7^ Delay can occur in the patient interval or the health systems interval.^8^ The patient interval refers to the time from the discovery of the first symptom to the first visit to a health care provider. Patient-related factors influencing delay include lack of awareness, fear of diagnosis or treatment, preference for spiritual or native care, and lack of funds, among others.^4-6,9-11^ The health system interval refers to the time from the first interaction with a health care provider until initiation of treatment.^8^ Provider-related factors contributing to delay include misdiagnosis, failure of appropriate referral, and incorrect treatment, among others.^4-6,9-11^

Prior research has identified major themes and factors linked to presentation delay.^4,9-11^ However, these reports vary widely, with some publications attributing the majority of delay to patient-related factors^12-14^ and others stating provider-related factors^15-18^ are the major contributor to the overall delay. Furthermore, the proportion of patients attributing their delay to different factors varies by publication and by country. For example, approximately 34% of women in Nigeria linked their delay to lack of awareness,^12^ whereas 66% of women in Zimbabwe linked their delay to lack of awareness.^14^ In a report by Clegg-Lamptey et al^16^ in Ghana, 11% and 20% of patients linked their delay to fear of diagnosis and the use of herbal medicine, respectively. Conversely, in a report by Otieno et al^13^ in Rwanda, 33% and 10% of patients linked their delay to fear of diagnosis and the use of herbal medicine, respectively.

Systematic reviews have been performed in which barriers to BC care in black patients and Africans were evaluated.^4,9-11^ The previous reviews narrated individual figures and ranked the factors on the basis of the number of articles mentioning each factor. However, to date, to our knowledge, no report has used meta-analysis to aggregate existing data and enable improved understanding of the distribution and the prevalence of themes linked to BC presentation delay in Africa. In addition, the prevalence of factors linked to delay is modifiable, and their distribution may change over time, between geographic regions, or after appropriate intervention.

With our meta-analysis, we aimed to (1) describe summary estimates of the prevalence and distribution of the themes and underlying factors linked to delayed presentation of BC in Africa, and (2) describe the 20-year trend of these themes and their regional variability. The rationale for this meta-analysis was to provide baseline estimates to assess the immediate situation, plan interventions, and track progress.

### METHODS

Using the Preferred Reporting Items for Systematic Reviews and Meta-Analyses (PRISMA) recommendations,^19^ a preliminary literature review, and needs assessment (using PubMed, Cochrane Library, and Prospective Register of Systematic Reviews [Prospero identifier CRD42019131361]) confirmed that a similar meta-analysis was not ongoing or conducted in the past 5 years.

We used the search terms identified in the preliminary review to conduct a full literature search in PubMed/Medline and African Journal OnLine between March 18, 2019, and July 2, 2019, as follows: “Delayed AND presentation AND breast cancer.” We conducted hand-searching in Google/Google Scholar, Science Direct, and ResearchGate and snowballing searches in the reference list of original articles and previous reviews.

### Article Screening

There were 2 levels of screening. The first was conducted by 1 author (A.O.) with Rayyan QCRI (https://rayyan.qcri.org/) using (1) date of publication, (2) article title, and (3) abstract review sequentially. The second was the full-text article review using predetermined Participant, Intervention, Comparison, Outcome, Time and Survey design (PICOTS) criteria, performed by 2 authors (A.O. and A.I.) with a third author (O.S.) acting as tie-breaker in cases of disagreement. The PICOTS criteria are as follows:

- Participants/population: Patient-reported surveys on factors perceived to be linked to delays in BC presentation in Africa. We excluded studies on delayed detection alone.
- Intervention and control: None.
- Outcome: Frequency count (quantitative data) of the themes and underlying factors linked to delay in presentation. We excluded studies from which the frequency count of the themes/factors could not be extracted and was not available after contacting the authors.
- Time: Surveys reported between January 1, 2000, and July 2, 2019, were reviewed.
- Study design: Cross-sectional, questionnaire-based, face-to-face survey case series with sample size ≥ 10; qualitative studies from which data on frequency of individual factors linked to the delay were available; and surveys of female patients with BC and mixed sexes in which male participation was < 10%. We excluded systematic reviews, case-control studies, case reports, and retrospective reviews. We also excluded studies focusing on specific factors (eg, delays linked to chemotherapy alone or linked to mastectomy alone).

In case of incomplete data leading to exclusion of publication, we contacted authors directly via e-mail to obtain unpublished data or gray literature and qualify the manuscript for inclusion.

### Data Extraction and Quality Assessment

Two authors (A.O. and A.I.) extracted the following data independently: study setting and design, the definition of delay, respondents’ age range, themes, and factors linked to delay. A third author (S.O.) resolved any disagreement. We adapted the quality assessment variables from the Strengthening the Reporting of Observational Studies in Epidemiology checklist.^20^ We used variables showing face, content, and construct validity for a questionnaire-based study focused on the factors linked to delay in presentation with BC. We placed a premium on representative data and transparent reporting. The 2 authors who extracted data independently allotted quality scores and discussed them to harmonize discrepancy.

### Classification of Factors for Analysis

For consistency, we adopted the 6 domains of themes and factors reported in the systematic review of reports from Africa^10^ as follows:

1. Symptom misinterpretation^21^: Factors linked to an error in judgment regarding the nature of the breast mass (eg, mischaracterizing malignant masses as benign) or underestimating the risk associated with the lesion (eg, thinking the breast mass was benign, associating the lesion with physiologic changes or delay in presentation because of lack of pain). We included ignorance or report of being unaware of BC under symptom misinterpretation on the premise that these patients did not interpret breast symptoms as cancer because they were not aware of BC.
2. Fear: Any mention of fear.
3. Preference for alternative care: Preference for unorthodox medical care such as spiritual care or traditional/native care.
4. Social: All social influences such as incorrect advice from relations, occupational demands, and keeping symptoms secret because of embarrassment.
5. Hospital: Direct hospital-related factors such as mistrust, misdiagnosis, inadequate treatment, strike (industrial) action in the health sector, or failure to refer.
6. Access: Distance, navigational or cost barriers.

To minimize questioner bias, only studies that reported frequency count (quantitative data) on at least 4 of the 6 themes were eligible. The distribution of the factors was not an exclusion criterion, because we recognized that specific factors varied with the environment.

### Statistical Analysis

The primary outcome was the proportion of each theme linked to delay in presentation. The themes and factors were evaluated as they related to the total interval (ie, the period between symptom detection and presentation in the tertiary care or specialist center), patient interval, or provider interval. The proportion of each theme or factor was calculated as follows: f = (frequency count of the specific theme or factor in the study) / (total frequency count of all themes / total frequency count of all factors reported in the study).

We estimated the following using meta-analytical procedure: (1) overall pooled prevalence and 95% CIs of the themes and factors linked to delay over 20 years (2000 through 2019); (2) the pooled prevalence and 95% CIs of factors linked to delay in the patient interval and the provider interval; and (3) subgroup analysis to show the temporal trend and explain regional variation.

We implemented the meta-analytical procedure in MetaXL add-in (http://www.epigear.com) for Microsoft Excel (Microsoft, Redmond, WA) with the quality-effect model, using the quality score derived for each study. Thus, we assigned higher weights to studies of better quality. An *I*^2^ > 75% indicated high heterogeneity. The pooled estimated of the prevalence was by the double arcsine transformation to avoid overweighting studies with values close to 0% or 100%. Subgroup analysis to explain trends was conducted on the basis of the year of publication from the years 2000 to 2010 (old) or after (recent). Subgroup analysis was also conducted to explain regional differences. The regional analysis included Eastern and Central Africa (ECA), Northern Africa (NA), West Africa (WA), and Southern Africa (SNA). We conducted the meta-analysis for regions for which there were at least 2 studies providing data.

## RESULTS

**FIG 1:**
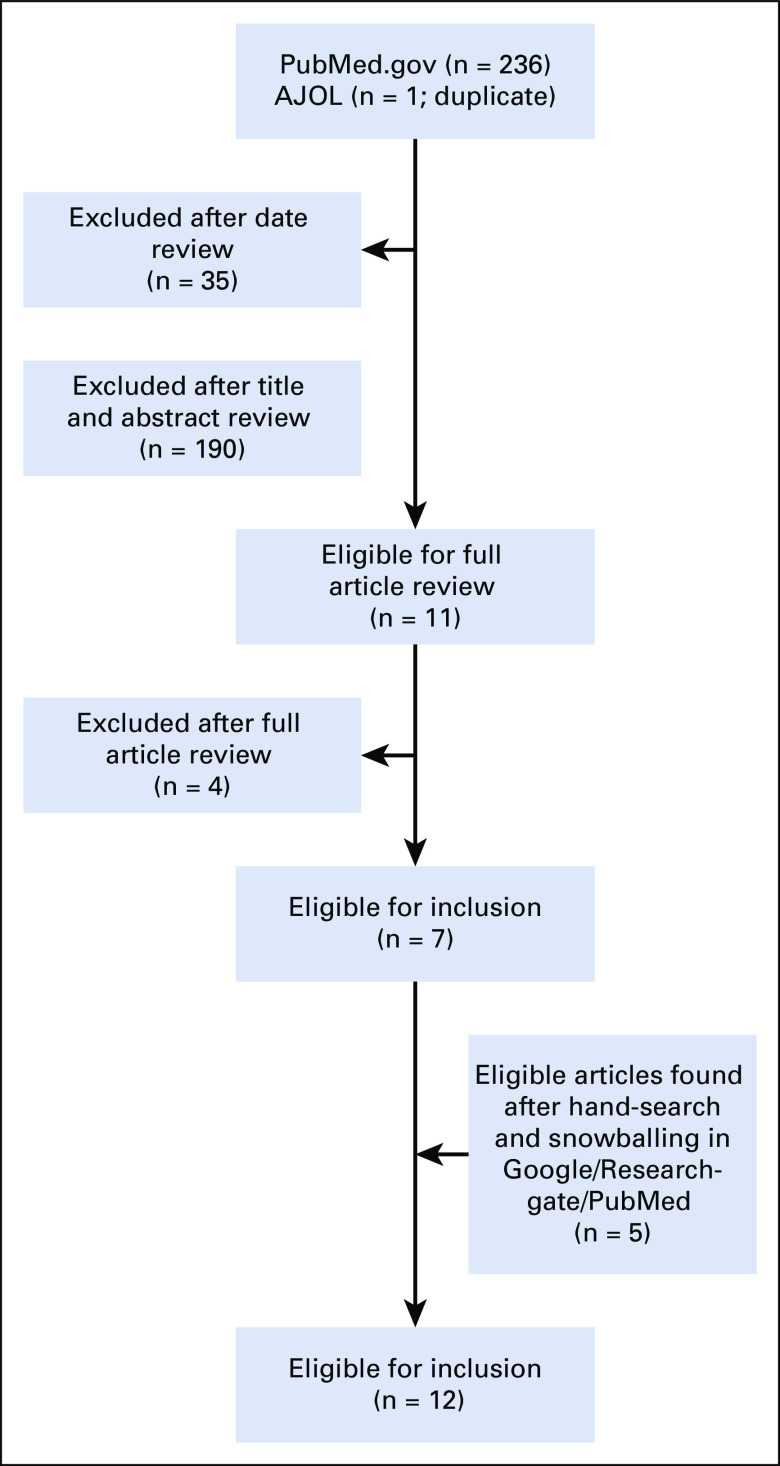
Preferred Reporting Items for Systematic Reviews and Meta-Analyses flow diagram showing article selection process. AJOL, African Journal OnLine.

The online search returned 236 citations (Fig 1). A total of 225 articles were excluded after date, title, and abstract review. Full-text review of the remaining publications revealed 7 that were eligible for inclusion. Five additional articles were eligible after hand-search and snowballing. Only 1 of the authors contacted by e-mail for relevant data responded, and the response did not yield additional data.^22^ This resulted in data from 12 publications being included in the meta-analysis (Table 1). Patient-level data regarding reasons for delay were available for 1,750 patients in the total interval, 456 in the patient interval, and 289 in the provider interval (Tables 2 and 3).

**TABLE 1:**
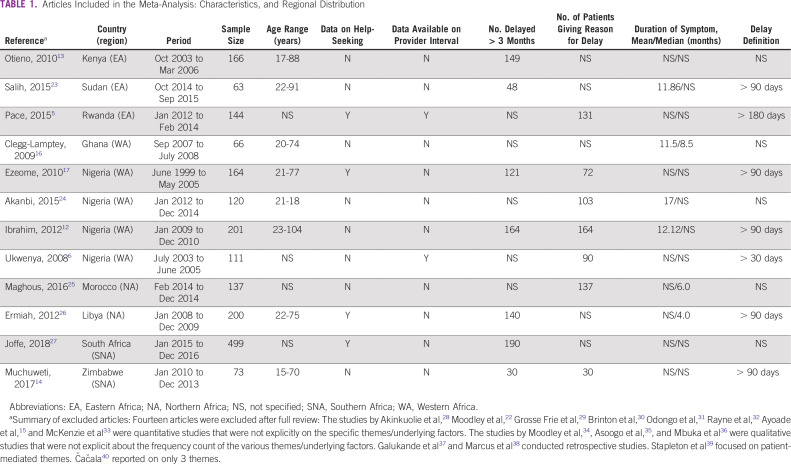
Articles Included in the Meta-Analysis: Characteristics, and Regional Distribution

**TABLE 2:**
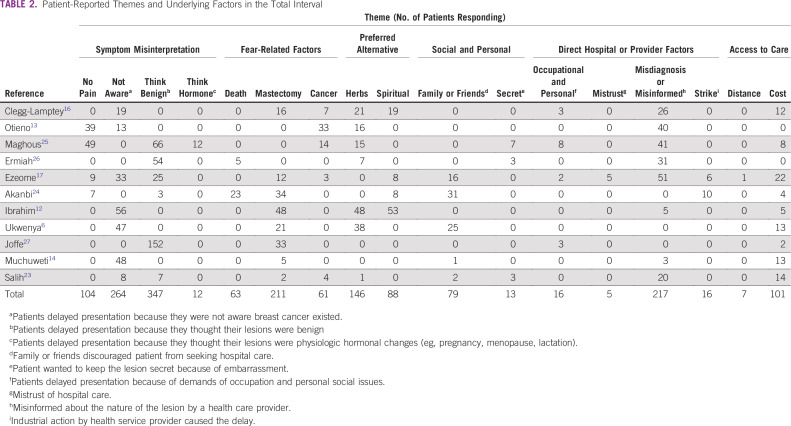
Patient-Reported Themes and Underlying Factors in the Total Interval

**TABLE 3:**
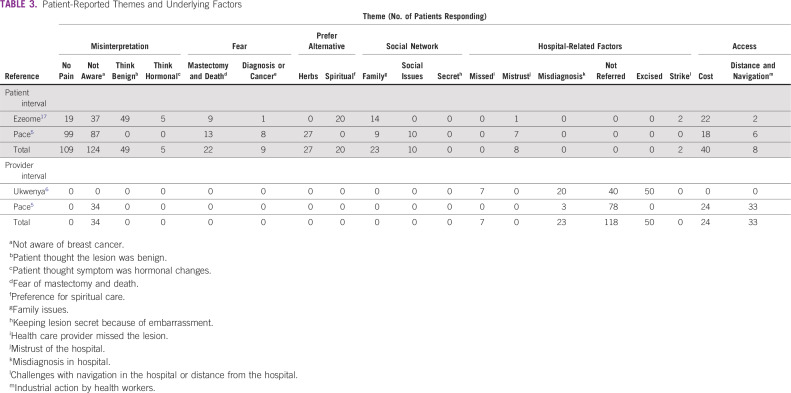
Patient-Reported Themes and Underlying Factors

### Quality Assessment

Eight articles omitted at least 1 variable and design characteristic that could improve the quality of their data, as shown in the quality assessment table in the Data Supplement. Not attempting to eliminate recall bias by triangulating or other means was the most common quality limitation. Five articles did not specify the number of respondents who delayed presentation or the number of respondents who provided reasons for their delay (Table 1). Two articles reported delays for stage III and IV disease only, perhaps assuming only patients with late-stage disease experienced delays in presentation.^13,25^

### Qualifying Delay

There were 2 patterns of reporting. In the first, the total delay was reported as a continuum (Table 2); in the second, the delay was reported in the component intervals: the patient interval and the provider interval (Table 3). The most common definition of total delay was an interval of > 90 days between symptom detection and arriving at a study center (Table 1). Six articles reported the mean or median duration of symptoms before presentation; the shortest was a median of 4 months, reported by Ermiah et al^26^ in Libya, and the longest was a mean of 17 months, reported by Akanbi et al^24^ in Nigeria (Table 1). The summary statistics showed 54% (95% CI, 23 to 84) of respondents experienced a delay > 90 days before arriving at a specialist clinic. The prevalence of delay was highest in ECA and WA (subgroup analysis of the prevalence of delay is reported in the Data Supplement).

### Summary Estimates of Factors Influencing Delay

Symptom misinterpretation was the most prevalent theme contributing to delays overall, accounting for 50% of all themes (Table 4). Fear-related factors (16%) were the next prevalent, followed by the hospital themes (11%) and preference for alternative care (10%). The influence of social factors and access-related issues were the least prevalent. Symptom misinterpretation dominated in the individual articles except in the study by Akanbi et al,^24^ where fear dominated, and in the report by Clegg-Lamptey et al,^16^ in which the hospital theme dominated. The error of thinking the lesion was benign (mischaracterizing symptoms as benign) was the most prevalent factor leading to symptom misinterpretation, and it accounted for twice as many delays as patients not being aware of BC (Table 4). Simple frequency count with descriptive proportion (based on only 3 reports, 2 of which were published before 2010) showed delay due to lack of awareness dominated in the patient interval, whereas failure to refer dominated in the provider interval (Table 3).

**TABLE 4:**
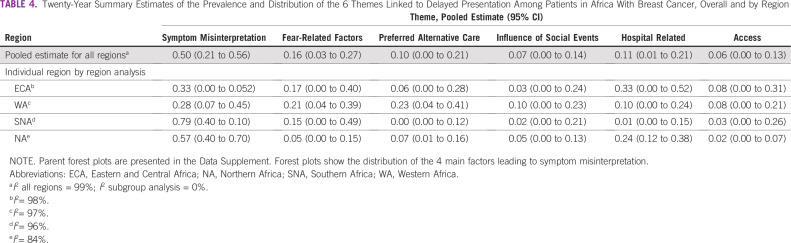
Twenty-Year Summary Estimates of the Prevalence and Distribution of the 6 Themes Linked to Delayed Presentation Among Patients in Africa With Breast Cancer, Overall and by Region

### Temporal Trends in the Distribution of Themes and Factors

**FIG 2:**
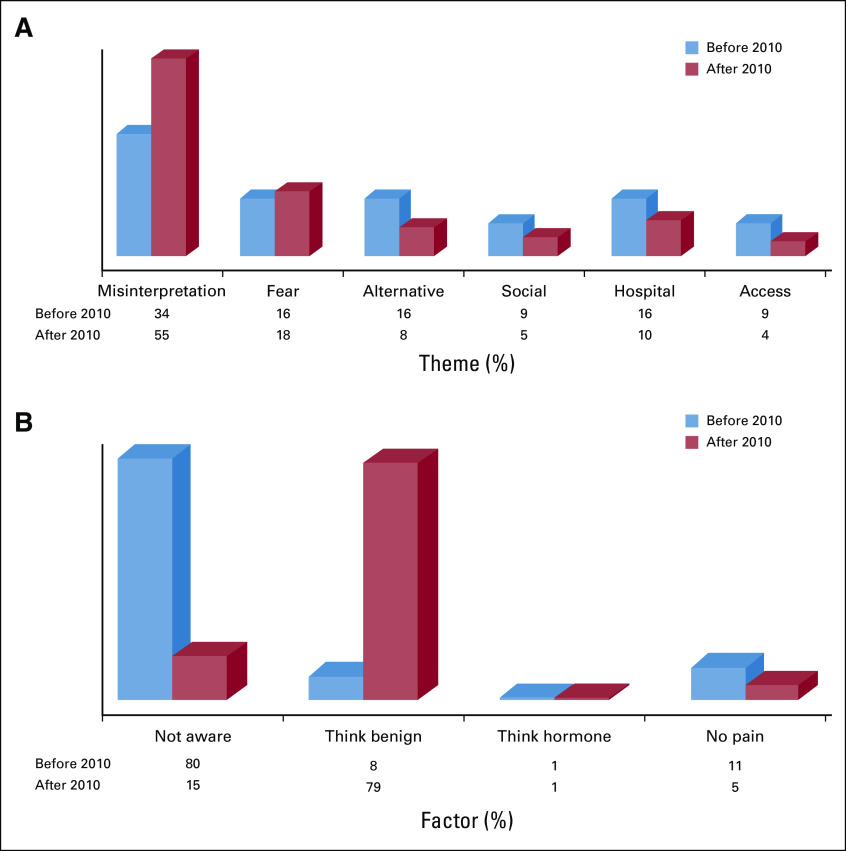
(A) Trend of themes contributing to delay before and after 2010. (B) Trend of the factors underlying symptom misinterpretation before and after 2010 (see parent forest plot in the Data Supplement). Access, access-related issues; Alternative, prefers alternative therapy such as native, spiritual, or herbs; Fear, fear of cancer or fear of treatment; Hospital, hospital-related issues; Misinterpretation, symptom misinterpretation; Not aware, not aware of breast cancer; No pain, delay due to experiencing no pain; Social, delay due social influence; Think benign, mistaking breast cancer for benign lump; Think hormone, attributing breast changes to hormonal changes.

The prevalence of all the themes was close to 10% or higher before 2010, with symptom misinterpretation being the most prevalent theme (34%). The prevalence of symptom misinterpretation became accentuated (55%) after 2010 (Fig 2). Lack of awareness was the dominant factor underlying symptom misinterpretation (80%) before 2010, but the error of mischaracterizing symptoms as benign (79%) overtook lack of awareness (15%) after 2010.

### Regional Variation in the Distribution of Themes and Factors

Symptom misinterpretation was the most prevalent theme in all regions (Table 4). The themes and factors underlying these themes were most diverse in WA and least diverse in SNA. The most prevalent themes in ECA and NA were symptom misinterpretation and hospital related. In WA, symptom misinterpretation and preference for alterative care were the most common themes. Symptom misinterpretation and fear-related themes were the most common themes in SNA.

Subgroup frequency distribution in the regions where the hospital-related theme was common (ie, ECA, WA, and NA) showed that misdiagnosis or misinformation by the health care provider was the most frequent contributing factor. The hospital-related factors were more diverse in WA, where failure to refer and treating the lesion by simple excision were common in the provider interval (Table 4). Subgroup frequency distribution in the regions where the fear-related theme was common (ie, WA and SNA) showed that concern for a mastectomy was the most prevalent fear.

Preference for herbal or native treatment was a more frequent reason for alternative treatment than spiritual care. The influence of advice from family or friends was the most prevalent social barrier, and the inability to afford the cost of treatment was the most prevalent access-related barrier (Table 3).

### Study Heterogeneity

There was marked heterogeneity in the overall summary estimates (*I*^2^ > 75%). The heterogeneity was eliminated, with *I*^2^ = 0 in the regional subgroup analysis (Data Supplement).

## DISCUSSION

We found through this meta-analysis that symptom misinterpretation was the most prevalent theme linked to delays in presentation among patients with BC in Africa, and the error of mischaracterizing symptoms as benign was the most common factor underlying symptom misinterpretation. Fear of mastectomy was the dominant fear-related factor. Misdiagnosis and failure to refer were the dominant hospital-related factors.

The themes linked to late presentation and their underlying factors are complicated in Africa and among black patients in developed countries.^4^ In a systematic review of factors contributing to late presentation of BC in Africa, Donkor et al^10^ found that negative symptom interpretation was a regional issue. Nearly all the studies they evaluated reported on fear, and they found that fear-related factors were the most significant factors linked to late presentation. This current meta-analysis corroborated that the factors linked to the delayed presentation are diverse, but contrary to Donkor et al.,^10^ our summary estimates showed that symptom misinterpretation is a continent-wide problem and its prevalence is much higher than fear-related themes, based on the weighted aggregates of individual figures.

When evaluating temporal trends in factors contributing to delays, we found that before 2010, lack of awareness of BC was the predominant factor contributing to symptom misinterpretation, but that factor was overtaken in the past 10 years by patients mistaking a malignant breast mass for a benign lesion. This shift is promising because it indicates an overall improvement in the awareness that BC exists. This may be due to intense campaigns to raise BC awareness that have become more common throughout Africa.^41^ Effective interventions must evolve as the burden of disease and barriers to care redistribute over time. Our findings elucidate a potential target for intervention in educating the general population about the signs and symptoms of BC and encouraging them to seek medical attention if symptoms develop. This provides hope for downstaging symptomatic disease in Africa.

Africa is diverse in its culture, the organization of its health care systems, and the challenges of BC management throughout the different regions.^2^ The incidence of BC is highest in SNA, but the socioeconomic burden and death rates are highest in the ECA and WA, as cited by Azubuike et al^42^ in their report on the global burden of BC with emphasis on sub-Saharan Africa. On the basis of these factors, we expected the burden of the themes or factors would be heterogeneous. However, the lack of heterogeneity in the regional analysis showed that countries in the same region may face relatively similar challenges. Part of the purpose for this research was to better understand diversity across regions to help plan situation-specific interventions. The variations in the themes and underlying factors reflected the facilities available and the health care systems adopted in the regions of Africa. Patient-related factors were dominant in NA and SNA, where the health care systems are relatively advanced and better organized compared with other regions. In contrast, factors in regions with less developed health care systems (ie, ECA, WA) were more diversity.

Aside from symptom misinterpretation, which was common to all regions, SNA had the fear-related theme as the other dominant issue. Fear of hospital treatment is one of the factors consistently linked to delayed presentation across Africa^32,42-44^ and is a prominent finding related to delay, even among black patients in developed countries.^4^ Fear of BC treatment can lead to delayed presentation with advanced disease, which often necessitates aggressive multidisciplinary treatment that is more difficult to tolerate, thus resulting in a vicious cycle. However, education regarding cancer diagnosis and effective interventions can help combat fear, promote earlier presentation, and, ultimately, allow for less morbid treatment.

In NA, the hospital-related theme was second to symptom misinterpretation and was most commonly due to misdiagnosis and failure to refer. Health care providers must be educated about triple assessment of breast symptoms: clinical review, radiologic evaluation and pathologic examination. Routine use of triple assessment can markedly increase the accuracy of diagnosis and should be performed even for lesions clinically diagnosed as benign to ensure accurate diagnosis, prompt referral, and effective treatment.

The access-related theme was high in WA and ECA, where the ratio of health care providers to patients is lowest.^41^ Surprisingly, pursuing alternative treatment was highest in WA, perhaps due to the ethnic diversity in that region. The preference for alternative care may also be due to the fear-related factors, which were high in WA and the false belief that alternative care is cheap. However, the same association did not appear in SNA, where fear-related factors were also high.

To our knowledge, this review is the first to use a meta-analytical method to describe the summary estimates of the themes and factors linked to delay in the presentation of BC in Africa, trends, and variation by region. The research gives insight into the continent-wide, patient-perceived challenges and regional differences by analyzing previously published data in a context that enables more impactful interpretation.

Strengths of this study include our rigorous review of existing published literature. Although the data collection in the original articles was not strictly quantitative, we included only quantitative data in our meta-analytical procedure. To limit intersurvey and interquestioner bias, we included only studies that reported on at least 4 of the 6 predetermined themes but allowed the factors to vary because we recognized that different environments might have different contexts.

The limitations of this study are similar to those of meta-analyses overall, which are subject to the limitations and quality of previously published data. Several of the articles reviewed were of low quality; nonetheless, we used the best data available to us on the subject. Moreover, the quality-effect meta-analytical model used here was an improvement over the random-effect model because it incorporated the heterogeneity in the quality of study design, thus giving more weight to studies with better quality. Future research studies should strive to conform to standard reporting in subintervals, as recommended in the Aarhus statement.^45^

Similar to previously published reviews, we faced the challenge of limited publications on this topic,^10,11^ and generally on BC in Africa. The continent-wide reviews by Espina et al^11^ included 21 articles, and Donkor et al^10^ included only 9 articles. We were able to locate all articles cited in these previous reviews, as well as additional reports through hand-searching and snowballing, because reports from Africa are not always published in high-impact or visible outlets. Furthermore, we have reported for subregions, but the situation may differ between countries even in the same region, depending on the economic and health care systems.

In conclusion, there are diverse themes underlying delay in presentation with BC in Africa, with marked regional variation. Modifiable factors, such as symptom misinterpretation, fear, misdiagnosis, and failure to refer are the most prevalent themes contributing to delays throughout Africa, and provide promising targets for future intervention
